# Mast cell-specific receptor MrgprB2 selectively mediates oxaliplatin-induced neuropathic pain

**DOI:** 10.1186/s12967-026-08285-w

**Published:** 2026-05-23

**Authors:** Leo C. Guan, Zhaoli Luo, Ruchita Kothari, Meilian Liu, Jieru Wan, Ankit Uniyal, Annie Y. Guan, Qing Lin, Xinzhong Dong, Jing Liu

**Affiliations:** 1 Brown University, Providence, RI USA; 2 Department of Anesthesiology and Critical Care Medicine, Johns Hopkins University School of Medicine, Baltimore, Maryland 21205 USA; 3 The Solomon H. Snyder Department of Neuroscience, Johns Hopkins University School of Medicine, Baltimore, MD USA; 4 Institute for Cell Engineering, Johns Hopkins University School of Medicine, Baltimore, MD USA; 5 McDonogh School, Owings Mills, MD USA; 6 Division of Cardiac Surgery, Department of Surgery, The Ohio State University College of Medicine, Columbus, OH USA; 7 Howard Hughes Medical Institute, Johns Hopkins University School of Medicine, Baltimore, MD USA

**Keywords:** MrgprB2/X2, Mast cells, Chemotherapy-induced peripheral neuropathy, Oxaliplatin, Pain

## Abstract

**Background:**

Chemotherapy-induced peripheral neuropathy (CIPN) causes debilitating pain that limits anticancer treatment, yet effective therapies remain limited. Mast cells (MCs) regulate neuroimmune signaling implicated in CIPN, but their receptor-specific contributions remain poorly understood. In mice, oxaliplatin and paclitaxel produce robust mechanical and cold hypersensitivities that mirror CIPN pain. *Mas*-related G-protein-coupled receptor B2 (MrgprB2) and its human orthologue MrgprX2 are MC-restricted receptors that mediate non-IgE-mediated activation. However, whether MrgprB2 signaling differently contributes to oxaliplatin- versus paclitaxel-induced CIPN pain remains unclear.

**Methods:**

We employed a comparative mechanistic approach using wild-type (WT) and MrgprB2 knockout (KO) mice to define receptor-specific contributions to oxaliplatin- versus paclitaxel-induced CIPN pain. Behavioral assessments of mechanical and cold hypersensitivity were integrated with histological profiling of MC recruitment and mediator profiles. Genetic findings were validated pharmacologically with osthole, a natural MrgprB2/X2 antagonist.

**Results:**

Both oxaliplatin and paclitaxel induced cutaneous MC accumulation in the hindpaw of WT mice, but they triggered distinct MC mediator-release profiles in vivo: oxaliplatin preferentially elevated tryptase, while paclitaxel was associated with greater histamine enrichment. Yet, neither drug directly activate MCs in vitro, as indicated by unchanged β-hexosaminidase release and calcium signaling. Importantly, MrgprB2 KO selectively attenuated oxaliplatin-induced tryptase upregulation and mechanical and cold hypersensitivity in vivo, without affecting paclitaxel-induced histamine changes or pain behavior. Consistently, with these genetic results, osthole selectively attenuated oxaliplatin-induced pain, but not paclitaxel-induced pain in WT mice.

**Conclusions:**

These findings support a mechanistic dichotomy in CIPN pain, indicating that MrgprB2 contributes selectively to oxaliplatin-induced, but not paclitaxel-induced, CIPN in mice. More broadly, they suggest that CIPN involves mechanistically distinct neuroimmune states depending on the chemotherapeutic class and highlight MrgprB2/X2 as a drug class-selective target for platinum-induced CIPN pain treatment.

**Supplementary information:**

The online version contains supplementary material available at 10.1186/s12967-026-08285-w.

## Background

Chemotherapy-induced peripheral neuropathy (CIPN) is a common, dose-limiting toxicity of anti-cancer medicines, affecting over 60% of patients receiving taxanes, platinum compounds, and vinca alkaloids [1–3]. The resulting symptoms, including cold allodynia, mechanical hypersensitivity, and chronic dysesthesia, often persist long after therapy ends, substantially impairing quality of life [1, 4]. Despite its high prevalence, therapeutic options remain critically limited, with standard-of-care drugs like duloxetine offering only modest benefits.

This therapeutic gap reflects, at least in part, a persistent “one-size-fits-all” view of CIPN, despite growing evidence that different chemotherapeutic agents engage partially distinct pathogenic mechanisms. Clinically, CIPN caused by platinum agents (e.g., oxaliplatin) and taxanes (e.g., paclitaxel) shares common features, manifesting as a length-dependent, “stocking-and-glove” distribution of sensory deficits, including numbness, tingling, and burning dysesthesias [5–7]. However, these symptomatic similarities may mask important mechanistic differences. Oxaliplatin is notable for acute cold hypersensitivity associated with ion channel dysregulation [8, 9], whereas paclitaxel-induced neurotoxicity is predominantly associated with microtubule disruption, mitochondrial dysfunction, and inflammatory signaling [6, 10]. These distinctions highlight the need to identify molecular effectors that help distinguish CIPN pain across chemotherapeutic classes.

Mast cells (MCs), positioned at the neuroimmune interface of the skin, are increasingly recognized as active contributors to peripheral pain sensitization [11–19]. Their granules contain multiple mediators that modulate nociceptive neuron excitability and local neuroinflammation [11, 20]. Crucially, the *Mas-*related G-protein-coupled receptor B2 (MrgprB2) in mice—and its functional human ortholog MrgprX2—are selectively expressed in MCs [16–19, 21]. Unlike classical IgE–FcεRI-mediated pathways, MrgprB2/X2 responds to a range of cationic secretagogues and neuroimmune mediators, including Compound 48/80, host defense peptides, and Substance P [18, 19, 22–25]. Because MCs can deploy distinct mediator-release programs depending on the nature and context of stimulation, MrgprB2/X2 is best viewed as a receptor that can preferentially engage certain MC activation states rather than as a marker of a single, pathway-exclusive secretory response [25]. This positions MrgprB2/X2 as a potential biologically specific target for chemotherapy drug class-selective treatment of CIPN pain. However, it remains unclear whether distinct chemotherapeutic agents differentially engage the MC-restricted MrgprB2/X2 pathway.

Prior work suggests that paclitaxel can increase Substance P release from cultured DRG neurons, whereas oxaliplatin does not, and paclitaxel neurotoxicity has also been linked to TLR4-dependent inflammatory signaling [26]. In contrast, the mechanism of platinum-induced neuro-immune crosstalk remains incompletely understood. Cisplatin, a related platinum analog, was shown to trigger the elevation of Substance P in dorsal root ganglion (DRG) tissues and promote neuro-immune interaction [27]. Based on this premise, we hypothesized that oxaliplatin and paclitaxel may differ in how they engage MC signaling during CIPN development, with oxaliplatin more likely to recruit a MrgprB2-dependent pathway in vivo [28–30], whereas paclitaxel may rely more heavily on MrgprB2-independent inflammatory mechanisms [31–33]. So far, neither how these agent-specific neuronal perturbations influence skin MC activation nor whether MrgprB2/X2 functions as a selective effector in platinum-induced CIPN pain has been tested.

Using complementary genetic, pharmacological, histological, and calcium-imaging approaches, we tested the hypothesis that oxaliplatin and paclitaxel may differ fundamentally in their mechanisms of engaging MC-associated signaling during the development of CIPN pain. Specifically, we asked whether oxaliplatin shows a greater dependence on MrgprB2 signaling than paclitaxel. Our findings may help to refine the mechanistic taxonomy of CIPN and highlight MrgprB2/X2 blockade as a promising translational strategy for alleviating oxaliplatin-induced CIPN pain.

## Materials and methods

### Animals

Adult C57BL/6J mice (8–10 weeks old, both sexes) were obtained from Jackson Laboratory. Transgenic lines, including MrgprB2 KO and MrgprB2-Cre; td-Tomato mice, were generously provided by Dr. Xinzhong Dong. Experiments were conducted using both male and female mice aged 8–12 weeks. Animals were housed in groups of 3 to 5 on a standard 12-hour light/12-hour dark cycle with free access to food and water. All use of animals was approved by the Animal Care and Use Committee of Johns Hopkins University and complied with the National Institutes of Health Guide for the Care and Use of Experimental Animals to ensure minimal animal use and discomfort. The transgenic animals created in our laboratory are available to the broader research community on receipt of a justified request.

### Osthole preparation and administration

A stock solution of osthole (O0426250MG, TCI America) was prepared at 500 mg/mL in DMSO. For each mouse, a 100 mg/kg dose of osthole was administered intraperitoneally (i.p.) using a formulation of 2% DMSO, 10% PEG400, 5% Tween-80, and normal saline. This dose was selected based on prior studies demonstrating its efficacy in blocking MrgprB2 activation [34]. Injections were given prior to each oxaliplatin or paclitaxel administration.

### Drug preparation and vehicle controls

For in vivo administration, oxaliplatin was prepared in 5% glucose at 0.3 mg/mL for injection. Paclitaxel was prepared at 0.8 mg/mL in a formulation containing Cremophor EL, ethanol, and 0.9% saline (5:5:90, v/v/v). Osthole was prepared in DMSO, PEG400, Tween-80, and normal saline (2:10:5:83, v/v/v/v). Corresponding vehicle-treated control groups were included for each in vivo paradigm.

For in vitro mast cell experiments, vehicle-matched controls were used for each drug condition. Oxaliplatin working solutions were freshly diluted from stock prepared in 5% glucose, and the matched vehicle control was generated using the corresponding dilution of the glucose vehicle. Paclitaxel working solutions were prepared by diluting the paclitaxel formulation, and the matched vehicle control contained the corresponding final dilutions of Cremophor EL, ethanol, and saline without paclitaxel. All in vitro drug effects were interpreted relative to their corresponding vehicle-treated controls within the same assay condition.

### CIPN models

WT and MrgprB2 KO mice were randomly assigned to oxaliplatin, paclitaxel, or vehicle (control) group, with a minimum of 8 mice per group for behavioral evaluation. Chemotherapy-related neuropathic pain was established by repeated intraperitoneal injections of oxaliplatin (O9512, Millipore Sigma, 3 mg/kg, i.p., twice weekly for 4 weeks) or paclitaxel (T7402, Millipore Sigma, 4 mg/kg, i.p., every other day for 4 times). These dosing regimens were selected strictly in accordance with established protocols validated to replicate the specific clinical pharmacokinetics and cumulative toxicity of each agent: the oxaliplatin protocol mimics the chronic cumulative neurotoxicity observed in the clinic [35], whereas the paclitaxel regimen follows the standard “dense-dose” model widely accepted for inducing robust, stable neuropathy with minimal systemic mortality [36, 37]. Despite differences in induction duration (chronic vs. subacute), behavioral evaluations and tissue collections were synchronized to occur during the maintenance phase of established neuropathy in both models: 4 weeks after the first oxaliplatin dose and 8 days after the first paclitaxel dose.

For MrgprB2 inhibitor intervention studies in WT mice, animals received osthole (100 mg/kg, i.p.) or vehicle (normal saline, i.p.) prior to each oxaliplatin or paclitaxel administration. Tissue samples were collected after completing CIPN induction, specifically, 4 weeks after the first oxaliplatin dose and 8 days after the first paclitaxel dose.

### Behavioral assays

The mechanical sensitivity of hind paws was evaluated using calibrated von Frey filaments (Stoelting, Wood Dale, IL, USA), following methods outlined in prior studies [38, 39]. Mice were placed on an elevated wire mesh platform within a clear plastic enclosure and acclimated to the testing environment for at least 30 minutes daily over 3 days before testing. A series of von Frey filaments (Stoelting Co.) (0.02, 0.04, 0.07, 0.16, 0.4, 0.6, 1.0, and 1.4 g) was used. Testing began with the 0.16 g filament, applied perpendicularly to the mid-plantar surface of either hind paw for 3–5 seconds. The 50% paw withdrawal threshold (PWT) was determined using the “up-and-down” method and calculated with the formula: $50\% \,{\mathrm{PWT}}\,(g)\, = \,\frac{{{{10}^{X\, + \,kd}}}}{{{{10}^4}}}$. X represents the value of the final filament used, k is the tabular value based on the pattern of positive/negative responses, and d is the average increment (in log units) between the filaments.

Cold sensitivity was evaluated using the cold-plantar test, which measured paw-withdrawal latency (PWL) in response to a dry ice pellet applied to a 6-mm-thick glass surface beneath the hindpaw [40]. A maximum cutoff time of 60 seconds was implemented to prevent tissue damage.

### Immunofluorescence

Adult mice were anesthetized with isoflurane in saline and transcardially perfused with 50 mL of phosphate-buffered saline (PBS) (114–058-101, Quality Biological), followed by 50 mL of 4% buffered paraformaldehyde solution (PFA) (1004960700, Millipore Sigma). Post-perfusion, the hind paw plantar skin was carefully excised and post-fixed in 4% PFA at 4 °C overnight, then transferred to 30% sucrose solution at 4 °C for 72 hours. Following sucrose infiltration, the tissue was embedded in optimal temperature compound (OCT) (Sakura Finetek) and stored at −80 °C until sectioning. The skin was sectioned at 20 μm thickness using a cryostat, and sections were collected onto slides. Sections were rinsed with PBS to remove residual OCT and incubated in a blocking solution (5% normal goat serum (G6767, Millipore Sigma) in PBS with 0.3% Triton-X 100 (X100, Millipore Sigma)) for 1 hour at room temperature. Primary antibodies were applied overnight at 4 °C in blocking solution (1% normal goat serum in PBS with 0.3% Triton-X 100), including: anti-tryptase (1:200; 13,343–1-AP, Thermo Fisher Scientific), anti-Histamine (1:200; PA5119564, Invitrogen), or anti-PGP9.5 (1:200; PA1-10011, Invitrogen). After primary incubation, sections were washed with PBS with Tween-20 (PBST) (3 times, 5 minutes each). Slides were subsequently rinsed with PBST (3 washes, 5 minutes each) and incubated with secondary antibodies diluted in blocking solution for 1 hour at room temperature. Secondary antibodies (Thermo Fisher Scientific) were used at 1:500. Slides were washed with PBST (5 times, 5 minutes each), mounted with Fluoromount-G containing DAPI (00–4959-52, Invitrogen), and imaged using a Zeiss LSM800 confocal microscope.

### Avidin staining protocol for mouse tissue

Avidin staining was performed by incubating tissue samples in blocking buffer for 3 hours at room temperature, followed by incubation with Avidin-Alexa Fluor FITC (Victorlabs) at a 1:300 dilution. Slides were subsequently rinsed with PBST (5 washes, 5 minutes each), mounted with Fluoromount-G containing DAPI (Thermo Fisher Scientific), and visualized using a Zeiss 800 confocal microscope.

### Mast cell degranulation quantification

Hindpaw plantar skin was collected from mice in each treatment group (3–6 per group), fixed, and processed for confocal imaging. Avidin-labeled mast cells were visualized using a laser-scanning confocal microscope under uniform acquisition settings. For each mouse, 3–7 non-overlapping fields were obtained from the plantar dermis of each hindpaw. Each field covered an area of 2.92 mm^2^. In each field, the total number of MCs and the number of degranulated MCs were counted manually. A MC was classified as degranulated when at least five extracellular granules were present within 5 μm of the cell membrane [41]. Granules were defined as discrete puncta (0.8–1.5 μm diameter) to minimize inclusion of background fluorescence. Counts from all fields for each mouse were summed to yield total MC and degranulated MC numbers. The percentage of degranulated MCs was calculated as: (number of degranulated MCs/total number of MCs) × 100%. All images were evaluated by investigators blinded to genotype and treatment.

### Mouse peritoneal mast cell (MPMC) culture

MPMCs were cultured as described [41, 42]. On day 1, the peritoneal cavity of 3 mice was flushed using ice-cold sterile PBS (10 mL), and the peritoneal cavity was gently massaged. The peritoneal fluid was then collected into a 50 mL centrifuge tube, and cells were spun (300×g, 10 min) followed by a PBS wash. Cells were re-suspended in 5 mL complete media (RPMI, 10% FBS (A567080, Thermo Fisher Scientific) /1% Penicillin-streptomycin (P4083, Millipore Sigma)) supplemented with recombinant mouse Interleukin-3 (rmIL-3) (10 ng/mL, I4144, Millipore Sigma) and Recombinant Mouse SCF (rmSCF) (30 ng/mL,455-MC, R&D Systems). The cell suspension was then transferred to a 25 cm^2^ tissue culture flask (P886-229341, Quality Biological) and incubated at 37 °C, 5% CO_2_. On day 3, non-adherent cells were discarded with the medium, and 5 mL of fresh medium containing rmIL-3 (10 ng/mL) and rmSCF (30 ng/mL) was added. On day 6, 5 mL of fresh media containing rmIL-3 (10 ng/mL) and rmSCF (30 ng/mL) was added to the culture. At the end of the culture (day 10), PMCs, the floating, non-adherent cells, were harvested at >95% purity (CD45+FcεR1+c-kit+), and the cells were used for the β-hexosaminidase assay.

### Beta hexosaminidase (β-hex) degranulation assay

MPMCs (50 μL of 2 × 10^6^ cells/mL in 10 mM HEPES+0.04%BSA, pH 7.4) from WT mice and MrgprB2 KO mice were seeded in a 96-well round-bottom plate. 50 μL of 2X treatment in warmed HEPES+BSA was then added to the cells, to obtain a final 1X concentration in the well [41]. MPMCs were treated with paclitaxel using an 8 μM final concentration, oxaliplatin using a 12 μM final concentration, or vehicle (HEPES+BSA). Cells were treated for 30 minutes at 37 °C and 5% CO2, then centrifuged at 300×g for 5 min. 50 μL of supernatant was removed and added to a 96-well assay plate. 50 μL of cell lysis buffer (0.1% Triton-X in PBS) was then added to the remaining cell and supernatant wells and mixed thoroughly. Once mixed, 50 μL of this solution was added to the other half of the 96-well assay plate. 50 μL of p-nitrophenyl N-acetyl-β-D-glucosaminide (487052, Sigma-Aldrich) in 0.1 M sodium citrate buffer (pH 4.5) was added to each well, and the mixture was incubated at 37 °C for 90 minutes. After incubation, the reaction was stopped by adding 50 μL of glycine buffer (0.4 M, pH 10.7) to each well. The plate was immediately read at absorbance 405 nm with 570 nm as a reference. The percentage of β-hexosaminidase released was calculated by the following formula:


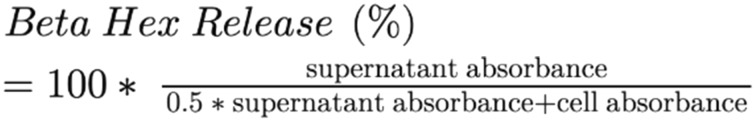


### Mast cell calcium imaging

Calcium imaging was performed using cultured MCs treated with vehicle-matched control, oxaliplatin (6 μM), paclitaxel (8 μM), or Compound 48/80 (10 μg/mL; positive control), across 32 wells (*n* = 8 per condition). For paclitaxel, the vehicle control contained the corresponding final dilution of Cremophor EL, ethanol, and saline without drug. Approximately 25,000 cells were plated per well from three independent cultures derived from seven mice, including both WT and MrgprB2 KO mice. Wells were pre-coated with 100 μL Poly-D-Lysine (A3890401, Thermo Fisher Scientific) at 37 °C and rinsed three times with sterile water. Cells were plated at 250 μL per well, centrifuged at 200 g for 2 min to promote adhesion, and incubated for 30 min at 37 °C in cell culture medium. Plates were organized as 4 × 4 wells per plate, with two plates representing the WT and MrgprB2 KO groups. Imaging was alternated between plates to minimize time outside the incubator while maximizing data acquisition. Calcium imaging followed the manufacturer’s Fluo-4 protocol (F10489, Thermo Fisher Scientific): cells were incubated for 1 hour in Fluo-4 solution at 37 °C, then imaged with a 100 ms exposure, 10% gain, and 13-second intervals for 5 minutes.

### Mast cell treatments and vehicle controls

Cultured MCs were used for in vitro degranulation, calcium imaging, and viability assays. Oxaliplatin [43] and paclitaxel [44, 45] concentrations used in the in vitro mast cell assays were chosen to fall within a short-term exposure range supported by prior in vitro pharmacokinetic studies. Their suitability under the present assay conditions was further supported by MTT and trypan blue viability testing, which showed no overt cytotoxicity at the working concentrations used in the manuscript. For all experiments, drug treatments were compared with their corresponding vehicle-matched controls. Oxaliplatin working solutions were freshly diluted from stock prepared in 5% glucose, and the matched vehicle control was generated using the corresponding dilution of the glucose vehicle. Paclitaxel working solutions were prepared by diluting the paclitaxel formulation, and the matched vehicle control contained the corresponding final dilutions of Cremophor EL, ethanol, and saline without paclitaxel. All in vitro findings were interpreted relative to the corresponding vehicle-treated control within the same assay condition.

### Calcium imaging data analysis

Photobleaching correction: Low-frequency baseline removal is standard practice in calcium-trace preprocessing pipelines [46, 47]. To correct for the effect of photobleaching as a result of light exposure during imaging, each trace *F(t)* was adjusted by removing baseline drift prior to normalization. This was achieved by fitting a cubic smoothing spline baseline *B(t)* to each trace *F(t)* in each data set, thereby isolating changes due to cellular activity from those resulting from fluorescent indicator degradation. Median *B(t)* is the median baseline value over the respective data set, and was then added back to each trace *F(t)* to retain activation scaling while correcting for photobleaching. The corrected signal was calculated through the equation:


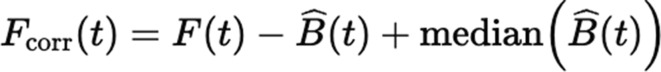


Pseudo activation filter: Sustained cytosolic Ca^2+^ elevation and loss of homeostasis are classical signatures of cell-death pathways [48, 49]. To avoid misclassifying non-physiological Ca^2 +^ overload as cellular activation, we quantified both the extent and duration of signal elevation following the peak, including short-term recovery within 60 seconds and long-term levels near the end of the recording. Traces exhibiting high, prolonged elevation or failing to recover, resulting in a persistent plateau, were flagged as cell-death pseudo-activation. Similarly, traces showing strong monotonic ramping without distinct peaks were classified as pseudo-activation. All remaining traces that satisfied detectability and recovery criteria were considered true activations. The formulas and equations used to calculate pseudo-activation are as follows:


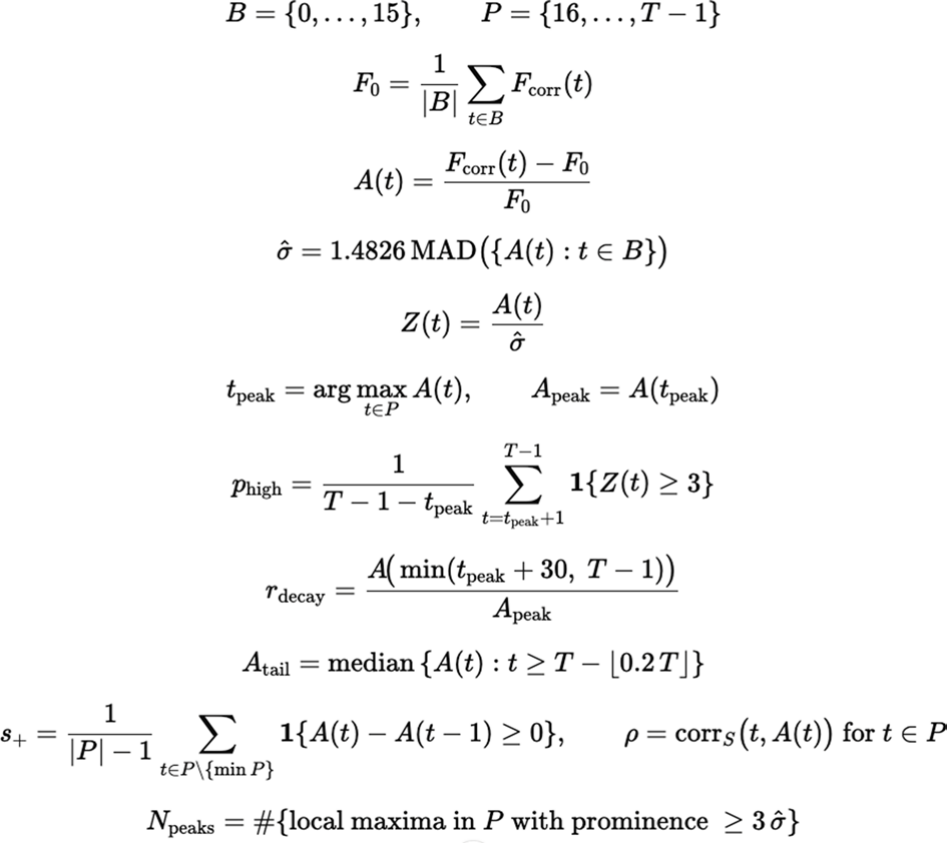


A trace is classified as cell-death pseudo-activation if it satisfies any of the following criteria:


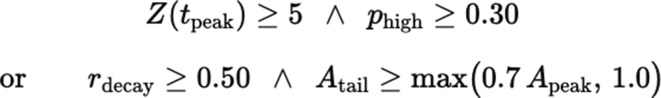


A trace is classified as monotonic-ramp pseudo-activation if it meets the following criteria:


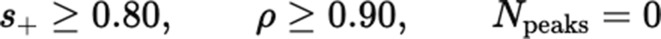


Normalization after correction: Normalization and computation of the primary response metric (*ΔF/F₀*) were performed after applying corrections for photobleaching, cell-death pseudo-activation, and monotonic-ramp pseudo-activation. The baseline *F*_*0*_ is defined as the mean of *F*_*corrected*_ (*F*_*corr*_) over 0–15 cycles of the pre-drug window. *F*_max_ is defined as the maximum value achieved during the post-drug window after 15 cycles. *ΔF* is defined as the *F*_max_ subtracted by the *F*_*0*_. Percent activation per data column was calculated through the following formula:


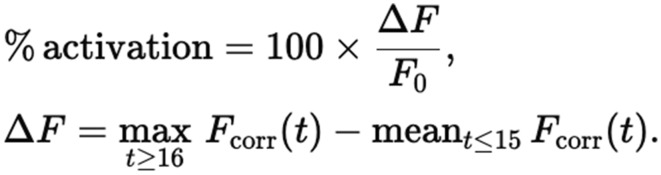


### MTT assay

Cell viability was evaluated using an MTT assay. Cultured MCs were seeded in 96-well round-bottom plates at 2 × 10^6^ cells/mL and exposed to oxaliplatin, paclitaxel, vehicle, or 0.1% Triton X-100 at 37 °C, 5% CO_2_. Drug exposure was performed for either 5 min, to match the acute calcium imaging condition, or 30 min, to match the pre-incubation condition. Cells were then washed once with fresh medium, transferred to flat-bottom 96-well plates, and incubated with MTT (475989, Sigma Aldrich) in fresh medium for 2 h at 37 °C. After centrifugation at 300×g for 5 min, the supernatant was removed and the pellet was dissolved in DMSO (D2438, Sigma Aldrich). Cells were transferred to flat-bottom 96-well plates. Absorbance was read at 570 nm. Blank wells without cells were used for background subtraction, and values were normalized to the corresponding vehicle control. Viability assays were performed under conditions matching those of the β-hexosaminidase degranulation assays and calcium imaging and, respectively. Blank wells containing medium and MTT but no cells were used for background subtraction, and viability was normalized to the corresponding vehicle-treated group. For each condition, viability was normalized to the corresponding vehicle-treated control.

### Trypan blue assay

Cell viability was further evaluated by trypan blue exclusion under the same treatment conditions used for the MTT assay. Cultured mast cells were plated in 96-well round-bottom plates at 2 × 10^6^ cells/mL and exposed to oxaliplatin, paclitaxel, vehicle, or 0.1% Triton X-100 for either 5 min or 30 min at 37 °C. Cells were then collected, washed once with fresh medium, and resuspended in fresh medium. A 10 μL aliquot of the cell suspension was mixed with 10 μL trypan blue, and the percentage of unstained cells among the total counted cells was measured using an automated cell counter-Countess™ 3 Automated Cell Counter (AMQAX2000, Invitrogen). Viability was assessed under conditions matched to those used for the β-hexosaminidase assays and calcium imaging, and normalized to the corresponding vehicle controls.

### Statistical analysis

ImageJ (NIH, USA) was used to analyze the images. The Prism 9.0 (GraphPad Inc., San Diego, CA) was used for all statistical analyses. Data were analyzed using one-way ANOVA with Tukey’s multiple comparisons test, two-way ANOVA with Tukey’s multiple comparisons test, or Student’s t-test as appropriate. Statistical significance was set at *p* < 0.05. Data are presented as mean ± SEM. Scattered individual plots were created using GraphPad Prism.

## Results

### Oxaliplatin and paclitaxel induced similar recruitment and degranulation of skin MCs

**Fig. 1:**
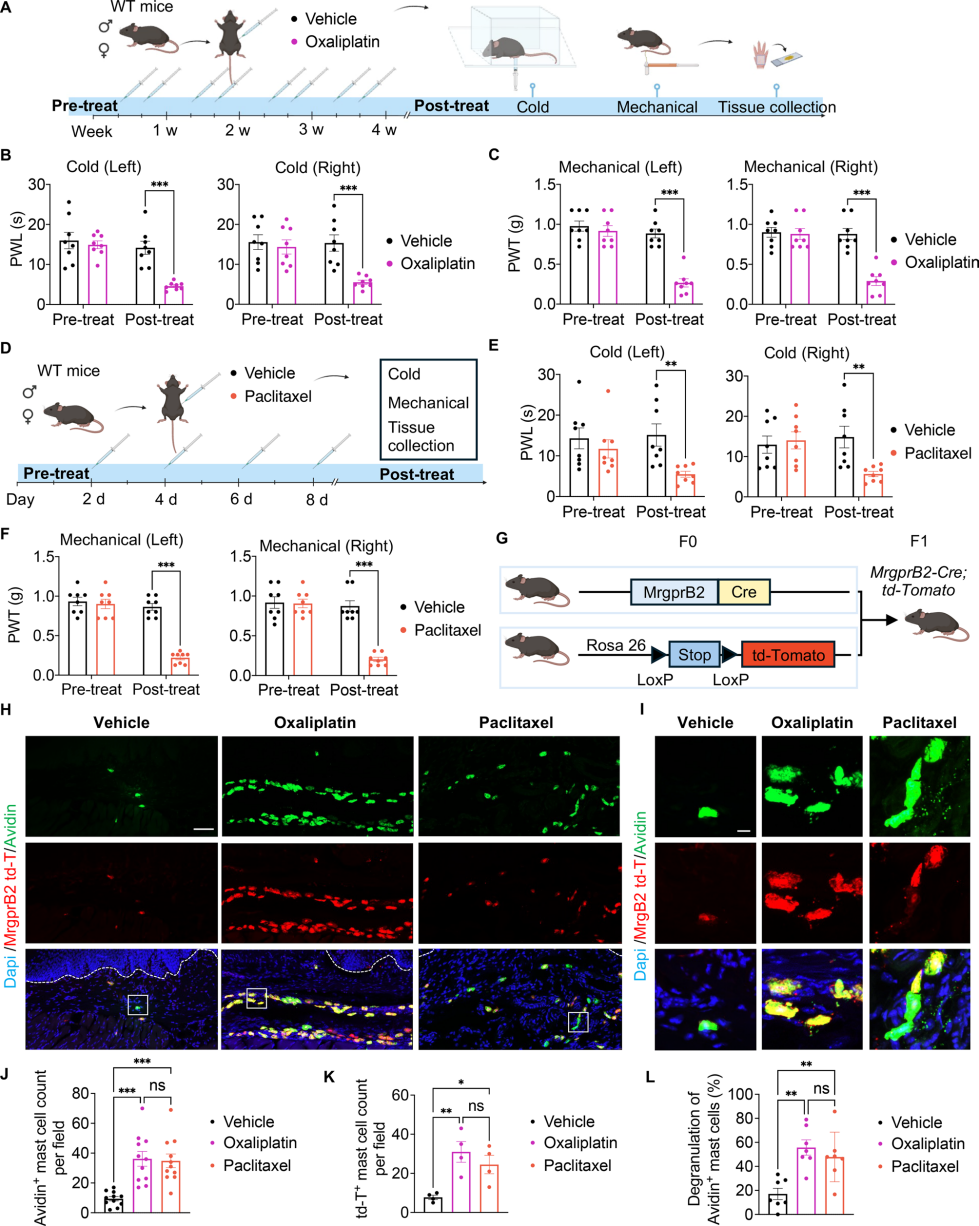
Both oxaliplatin and paclitaxel induced CIPN pain and increased mast cell recruitment and degranulation in the skin. (**A**) Outline of experimental strategy for oxaliplatin-induced CIPN. (**B**) Cold hypersensitivity assessed by paw-withdrawal latency (PWL) in the bilateral hindpaws was assessed using the dry ice test. (**C**) Mechanical hypersensitivity, evaluated by paw-withdrawal threshold (PWT), was assessed using the von Frey filament test, revealing reduced mechanical thresholds following oxaliplatin treatment compared with vehicle controls. (**D**) Outline of the experimental strategy for paclitaxel-induced CIPN. (**E**) Cold hypersensitivity in the hindpaw was assessed using the dry ice test, and (**F**) mechanical hypersensitivity was examined with von Frey filament testing. B, C, E and F: *n* = 8/group, data are represented as mean ± SEM **p < 0.01, ***p < 0.001; one-way ANOVA with Tukey’s multiple comparisons test (*n* = 8/group). (**G**) Strategy for generating MrgprB2-Cre; td-Tomato mice. (**H**) Representative confocal immunofluorescence images and (**I**) magnified view of hindpaw plantar skin showing avidin (green, marking degranulated mast cells), tdT positive (^+^) mast cells (red), and Dapi (blue, nuclei). (**J**) Quantification of Avidin^+^ mast cells per field (*n* = 11/group). (**K**) Quantification of tdT^+^ mast cells per field (*n* = 4/group). (**L**) Percentage of degranulated Avidin^+^ mast cells (*n* = 4/group). Scale bar: 200 μm in (H) and 20 μm in (I). J, K and L: *n* = 4–11/group, data are mean ± SEM. *p < 0.05, **p < 0.01, ***p < 0.001; one-way ANOVA with Tukey’s multiple comparisons test (*n* = 4–11/group)

Oxaliplatin treatment in WT mice led to cold, as measured by dry ice testing, and mechanical allodynia, assessed using von Frey filaments, with thresholds significantly decreased relative to the vehicle-treated group (Fig. 1A–C). Paclitaxel treatment resulted in a similar behavioral profile, with significantly reduced mechanical thresholds and increased cold sensitivity compared to vehicle control (Fig. 1D–F).

By crossing MrgprB2-Cre mice with td-Tomato (tdT) reporter mice, MrgprB2-positive (^+^) cells can be readily identified by red fluorescence (Fig. 1G). In MrgprB2-Cre;td-Tomato mice, confocal imaging of hindpaw skin revealed co-localization of tdT signal with the degranulated MCs labeled with avidin, a well-established marker to demonstrate MC morphology [50, 51], confirming that MrgprB2 is expressed in skin MCs (Fig. 1H, I). According to the published literature, the MrgprB2-Cre;td-Tomato+ labeling is restricted to MCs and shows near-complete colocalization with avidin [16, 41, 52]. Compared with vehicle treatment, both oxaliplatin and paclitaxel significantly increased the number of avidin^+^ and td-T^+^ MCs in the skin (Fig. 1J, K), and the percentage of MCs showing degranulation (Fig. 1L). Direct statistical comparison between the oxaliplatin and paclitaxel groups did not reveal a significant difference in any of these parameters. Thus, although the two drugs diverged in downstream mediator profiles, their effects on MC recruitment and degranulation were not significantly different at this level of analysis.

### Oxaliplatin and paclitaxel produced differential changes in tryptase and histamine expression in MCs

Tryptase and histamine releases are established markers of MCs activation, but their relative release can vary across activation contexts rather than mapping exclusively onto a single signaling pathway [19, 25]. We therefore conducted immunostaining to compared changes in tryptase and histamine expression in the skin MCs of MrgprB2-Cre;td-Tomato mice after drug treatment. Because oxaliplatin and paclitaxel CIPN models differ in induction duration, with tissue collection performed at the maintenance phase within each paradigm rather than at identical elapsed times, these comparisons should be interpreted accordingly.

**Fig. 2:**
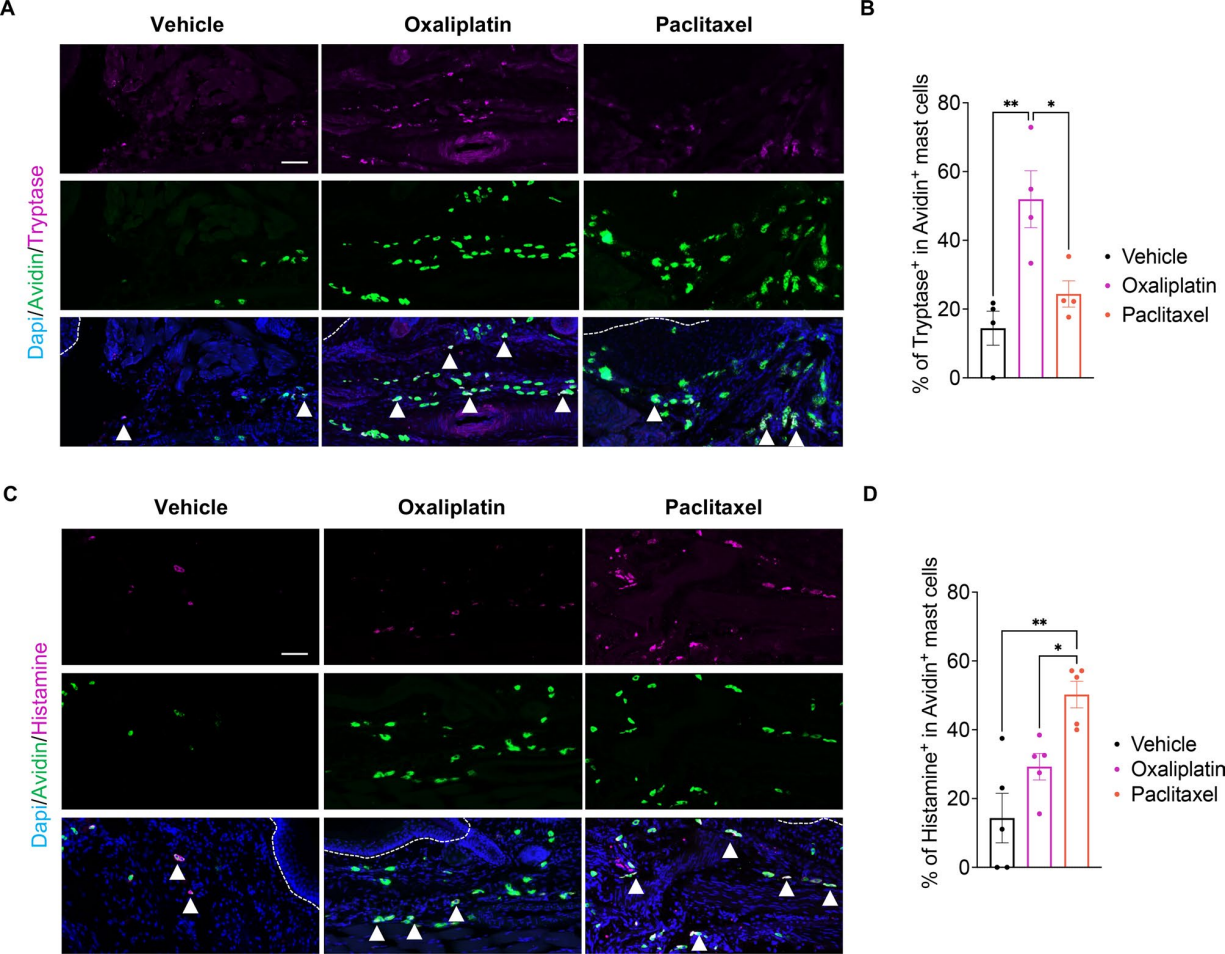
Oxaliplatin preferentially increased tryptase, whereas paclitaxel preferentially elevated histamine in hindpaw skin. (**A**) Representative confocal immunofluorescence images of hindpaw skin sections stained for tryptase (magenta), avidin (green), and Dapi (blue; nuclei). White arrowheads heads show co-localization. (**B**) Quantification of tryptase^+^/avidin^+^ cells per field (*n* = 4/group). (**C**) Representative images as in (A) but stained for histamine (magenta) with avidin and Dapi. (**D**) Quantification of histamine^+^/avidin^+^ cells per field (*n* = 5/group). Scale bar, 200 μm. Data are mean ± SEM. *p < 0.05, **p < 0.01; one-way ANOVA with Tukey’s multiple comparisons test

In hindpaw skin, oxaliplatin significantly increased the number of tryptase^+^ /avidin^+^ double-labeled MCs compared to vehicle controls, whereas paclitaxel induced a significantly smaller change (Fig. 2A, B). By contrast, histamine staining showed only a modest increase after oxaliplatin treatment, whereas paclitaxel induced a more pronounced elevation, with significantly more histamine^+^ /avidin^+^ double-labeled MCs per field than the oxaliplatin group (Fig. 2C, D). Thus, within the respective maintenance phases of these two CIPN models, oxaliplatin and paclitaxel were associated with distinct mast cell mediator profiles in the skin.

### MrgprB2 deletion selectively reduced oxaliplatin-induced pain and MC activation

**Fig. 3:**
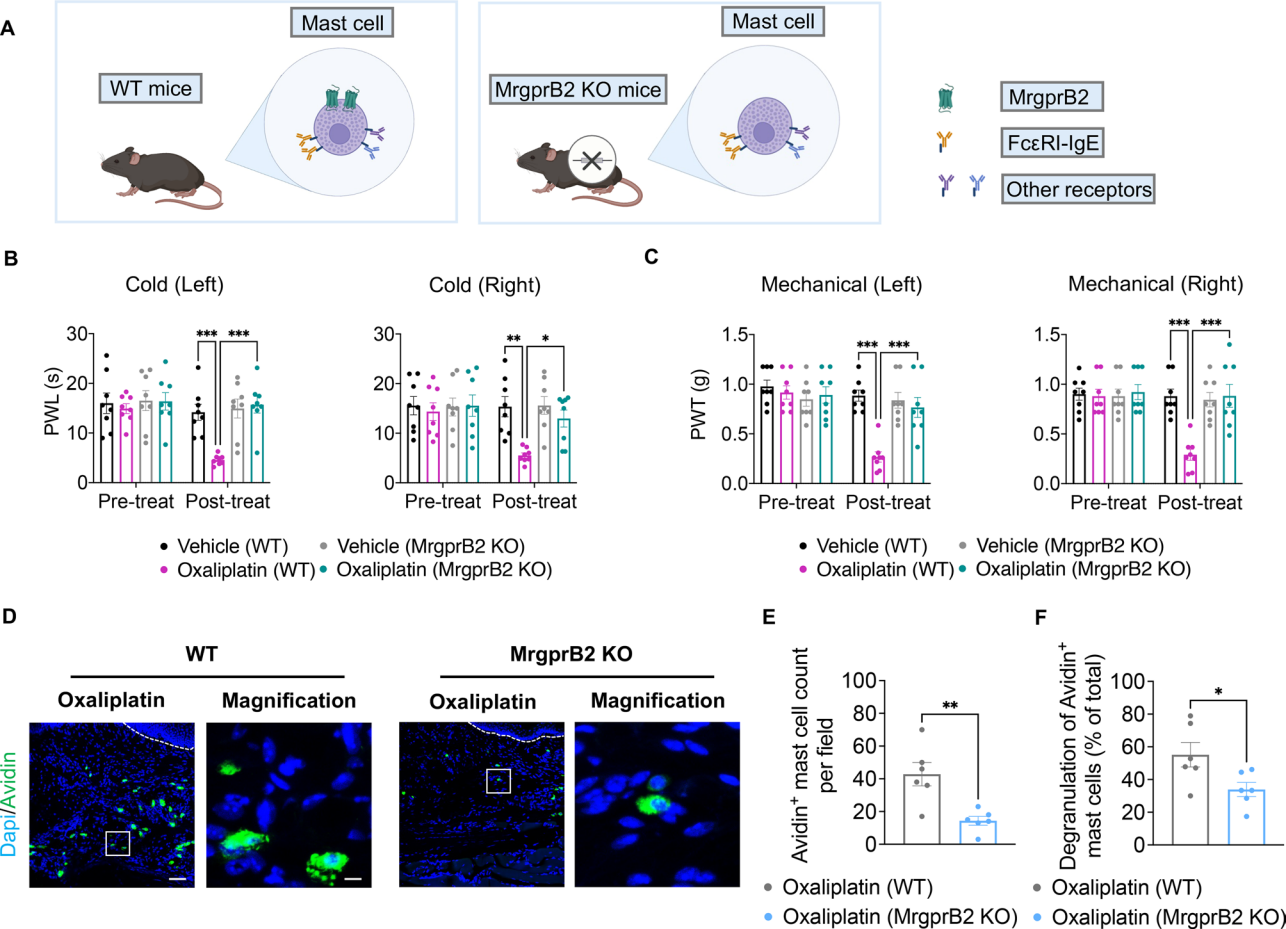
Knockout (KO) of MrgprB2 attenuated oxaliplatin-induced cold and mechanical hypersensitivities and reduced mast cell recruitment in the skin. (**A**) Schematic diagram of receptor expression on mast cells in wild-type (WT) mice and MrgprB2 KO mice. (**B**) PWL to cold stimuli and (**C**) PWT to mechanical stimulation in WT and MrgprB2 KO mice before and after oxaliplatin treatment (*n* = 8/group). Data are presented as mean ± SEM. *p < 0.05, **p < 0.01, ***p < 0.001, two-way ANOVA with Tukey’s multiple comparisons test. WT mice data were re-plotted for comparison. (**D**) Left: Representative confocal immunofluorescence images of hindpaw skin sections stained for avidin (green) and Dapi (blue). Scale bar, 200 μm. Right: higher magnification of boxed area. Scale bar, 20 μm. (**E-F**) Quantification of avidin^+^ mast cells (E) and percentage of degranulated mast cells (F) per field in MrgprB2 KO and WT mice after oxaliplatin treatment (*n* = 6/group). Data are mean ± SEM. *p < 0.05, **p < 0.01, student’s t-test

To examine the functional role of MrgprB2 in CIPN pain, we measured cold and mechanical hypersensitivities, two hallmark features of CIPN, in MrgprB2 KO and WT mice treated with oxaliplatin, paclitaxel, or vehicle. Deletion of MrgprB2 significantly attenuated oxaliplatin-induced mechanical and cold hypersensitivity, compared to that in WT mice (Fig. 3A–C). The PWL and PWT were not significantly altered after vehicle treatment in either genotype. In the hindpaw skin, oxaliplatin significantly increased the number of avidin^+^ MCs and the proportion of degranulated MCs in WT mice, compared to vehicle (Fig. 1J, L). However, both measures were significantly reduced in MrgprB2 KO mice, compared with those in WT mice (Fig. 3D–F).

**Fig. 4:**
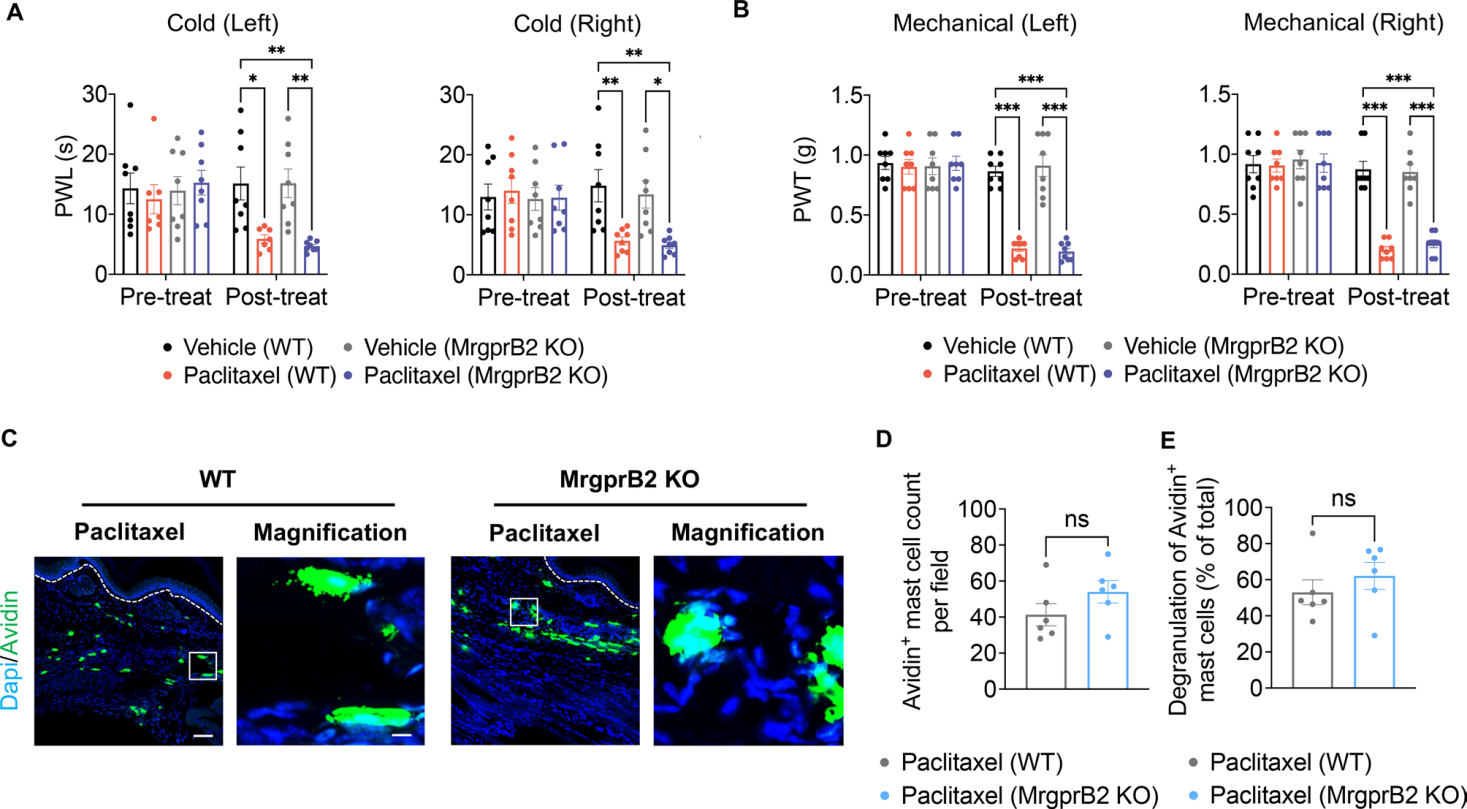
MrgprB2 deletion did not prevent the development of cold and mechanical hypersensitivity induced by paclitaxel. (**A-B**) Paw-withdrawal latency (PWL) to cold stimuli (A) and paw-withdrawal threshold (PWT) to mechanical stimulation (B) in WT and MrgprB2 KO mice before and after paclitaxel treatment (*n* = 8/group). Data are presented as mean ± SEM. *p < 0.05, **p < 0.01, ***p < 0.001, two-way ANOVA with Tukey’s multiple comparisons test. WT mice data were replotted for comparison. (**C**) Left: Representative confocal immunofluorescence images of hindpaw skin sections stained for avidin (green) and Dapi (blue). Scale bar, 200 μm. Right: higher magnification of the boxed area. Scale bar, 20 μm. (**D-E**) Quantification of avidin^+^ mast cells (D) and percentage of degranulated mast cells (E) per field in MrgprB2 KO and WT mice after paclitaxel treatment (*n* = 6/group). Data are mean ± SEM, student’s t-test

Unlike oxaliplatin, both genotypes showed comparable cold and mechanical hypersensitivities after paclitaxel treatment (Fig. 4A, B). The PWL and PWT were not significantly altered after vehicle treatment in either genotype. Paclitaxel also produced a similar increase in the number of avidin^+^ MCs and the proportion of MC degranulation in MrgprB2 KO mice as observed in WT mice (Fig. 4C–E). These findings suggest that MrgprB2 may contribute to oxaliplatin-induced behavioral pain hypersensitivity and MC recruitment and activation in the skin. However, it may be dispensable for the development of paclitaxel-induced pain and MC activation.

### Pharmacological blockade of MrgprB2 also attenuated oxaliplatin-induced pain

**Fig. 5:**
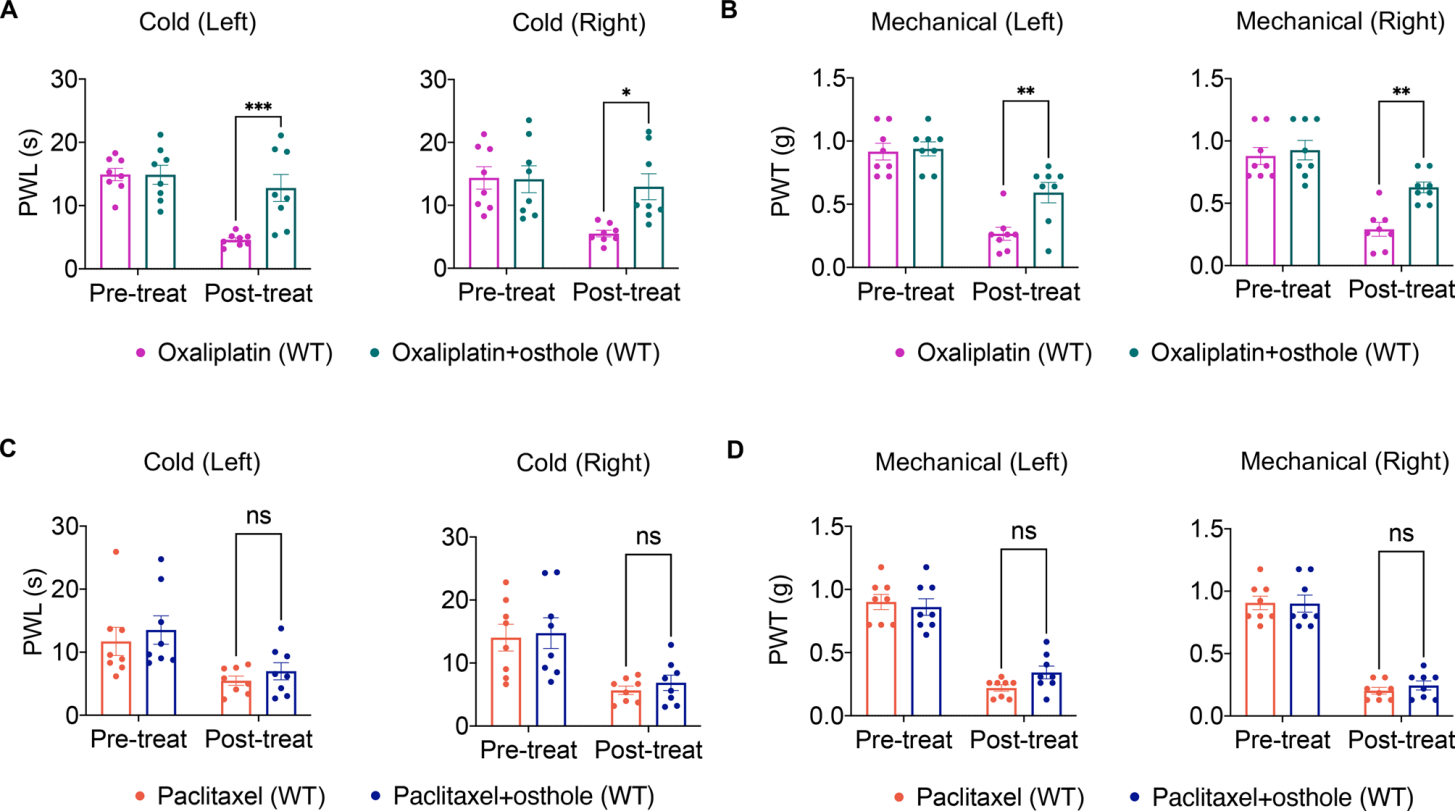
Pharmacological inhibition of MrgprB2 reduced both cold and mechanical hypersensitivity induced by oxaliplatin but had no effect on those induced by paclitaxel. (**A-B**) Paw-withdrawal latency (PWL) to cold stimuli (A) and paw-withdrawal threshold (PWT) to mechanical stimulation (B) in WT mice before and after oxaliplatin treatment with intraperitoneal injection (i.P.) of osthole (100 mg/kg) or vehicle co-treatment (*n* = 8/group). (**C-D**) PWL to cold stimuli (C) and PWT to mechanical stimulation (D) before and after paclitaxel treatment in WT mice with osthole (100 mg/kg, i.P.) or vehicle co-treatment (*n* = 8/group). Data are mean ± SEM. *p < 0.05, **p < 0.01, ***p < 0.001, two-way ANOVA with Tukey’s multiple comparisons test. Data from WT mice treated with oxaliplatin or paclitaxel were replotted for comparison

Osthole is a natural MrgprB2/X2 antagonist and has been used to examine the functional role of MrgprB2/X2 signaling [34, 41, 53]. During oxaliplatin-induced CIPN pain induction in WT mice, co-administration of osthole significantly attenuated both mechanical and cold hypersensitivity compared with vehicle co-treatment (Fig. 5A, B). In contrast, paclitaxel-induced pain hypersensitivity developed similarly in mice receiving osthole or vehicle co-treatment (Fig. 5C, D). These findings are consistent with MrgprB2 KO data and indicate that pharmacological inhibition of MrgprB2 also selectively mitigates oxaliplatin-induced CIPN pain.

Because both male and female mice were included in this study, we further performed an exploratory sex-disaggregated analysis of the behavioral datasets. When the pooled data were separated by sex within the same experimental panel, the overall pattern of treatment and genotype effects was similar to that observed in the pooled analyses, without an obvious sex-dependent divergence (Figs. S1, S2). Specifically, paclitaxel-induced mechanical hypersensitivity developed in both male and female mice, and MrgprB2 deficiency did not produce a clear sex-specific alteration in this response. Nevertheless, these analyses were not powered for formal inference regarding sex-specific effects and are therefore presented as exploratory.

### Oxaliplatin and paclitaxel did not directly activate MCs nor enhance the calcium response of MC to Substance P and S1P

**Fig. 6:**
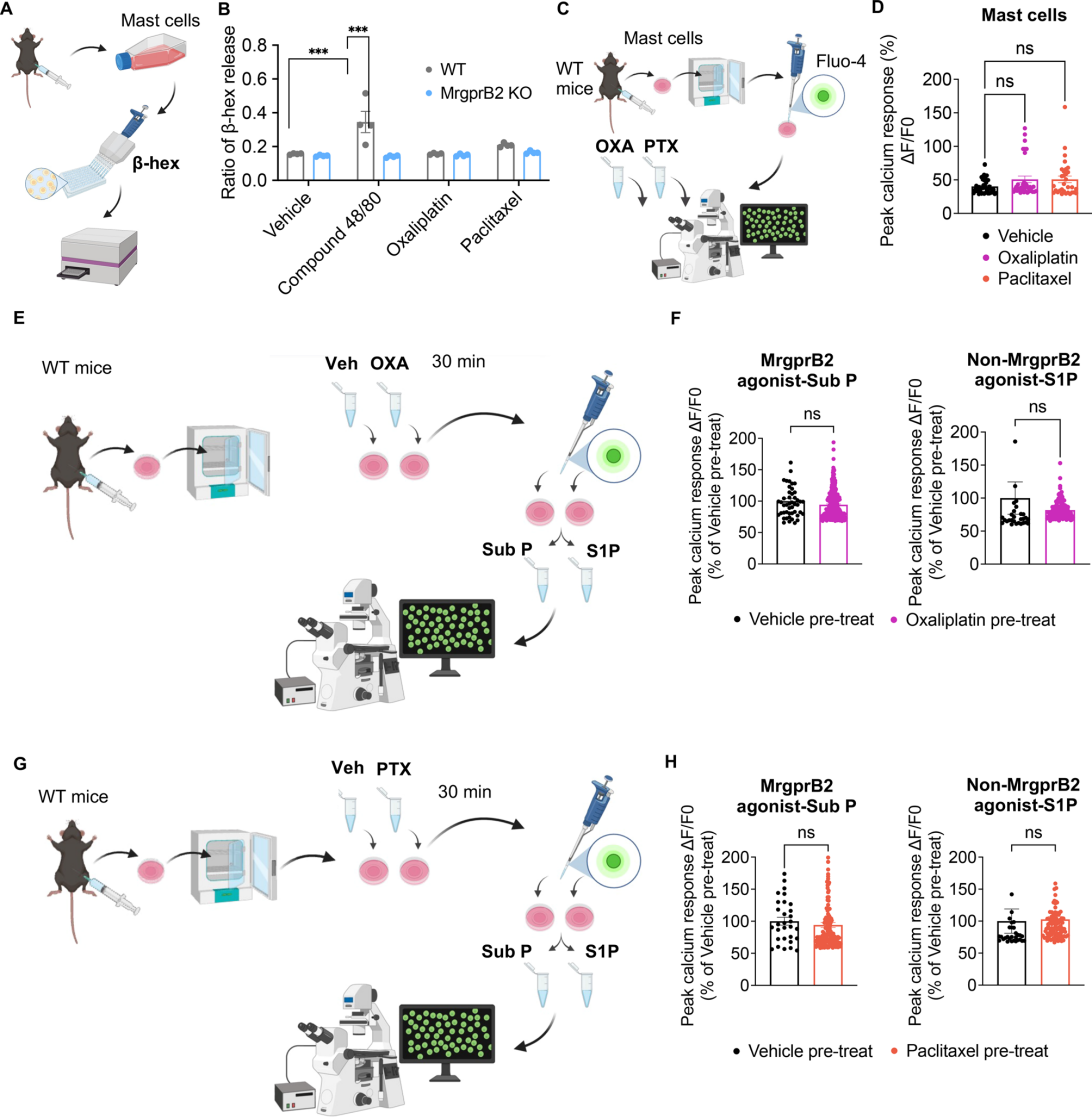
Neither oxaliplatin nor paclitaxel directly triggers mast cell degranulation or calcium response in vitro. (**A**) Schematic diagram illustrating the experimental setup for β-hex assays in cultured mast cells. (**B**) Quantification of β-hex release following treatment with vehicle, compound 48/80 (MrgprB2 agonist, a positive control) (10 μg/mL), oxaliplatin (12 μM) or paclitaxel (8 μM). Data are mean ± SEM. *** *p* < 0.001, one-way ANOVA with Tukey’s multiple comparisons test (*n* = 4/group). (**C**) Schematic of the in vitro calcium imaging workflow in cultured mast cells. (**D**) Quantification of in vitro calcium responses. Data are mean ± SEM. One-way ANOVA with Tukey’s multiple comparisons test (*n* = 31–47 cells/group). (**E**) Schematic of in vitro calcium imaging of cultured mast cells with OXA (oxaliplatin) preincubation for 30 mins. (**F**) Quantification showing that oxaliplatin preincubation did not alter calcium signals in mast cells evoked by sub P (Substance P, MrgprB2-dependent) and S1P (MrgprB2-independent), compared with vehicle preincubation. (**G**) Schematic of in vitro calcium imaging of cultured mast cells with PTX (paclitaxel) preincubation for 30 mins. (**H**) Quantification showing that paclitaxel preincubation did not alter sub P- and S1P-evoked calcium signals in mast cells compared with vehicle. F and H: data are mean ± SEM. One-way ANOVA with Tukey’s multiple comparisons test (*n* = 29–362 cells). Vehicle-matched controls were used for each drug condition. The paclitaxel vehicle consisted of the corresponding dilution of the Cremophor EL/ethanol/saline formulation without paclitaxel

Direct application of oxaliplatin or paclitaxel into the culture medium did not induce MC degranulation in WT mice and MrgprB2 KO mice in vitro, as indicated by a lack of increase in β-hexosaminidase release, compared to vehicle (Fig. 6A, B). In contrast, application of compound 48/80, which is known to activate MrgprB2, produced a significant increase in β-hexosaminidase release from cultured MCs from WT mice, but not MrgprB2 KO mice (Fig. 6B).

Moreover, in calcium imaging, neither chemotherapy agent induced an intracellular calcium rise in cultured MCs from WT mice compared with vehicle (Fig. 6C, D). Compared with vehicle-retreatment, preincubation of MCs from WT mice with oxaliplatin for 30 minutes failed to alter subsequent calcium responses of MCs to Substance P and S1P, which evoke MrgprB2-dependent and independent calcium rises, respectively (Fig. 6C, D). In addition, Substance P-induced and S1P-induced calcium responses in MCs were also not unaffected by preincubation with paclitaxel (Fig. 6E, F).

To determine whether these negative findings may result from nonspecific cytotoxicity induced by oxaliplatin and paclitaxel, we assessed MC viability under conditions matched to the functional assays, including the 5 min and 30 min calcium imaging paradigms and the 30 min β-hexosaminidase degranulation condition. Oxaliplatin and paclitaxel did not reduce MC viability at the concentrations tested in these assay conditions (Fig. S3). While the higher concentrations of oxaliplatin and paclitaxel, and Triton X-100 (a positive control), reduced MC viability (Fig. S3).

### MrgprB2 selectively mediates oxaliplatin-induced tryptase upregulation but not paclitaxel-driven histamine elevation in vivo

**Fig. 7:**
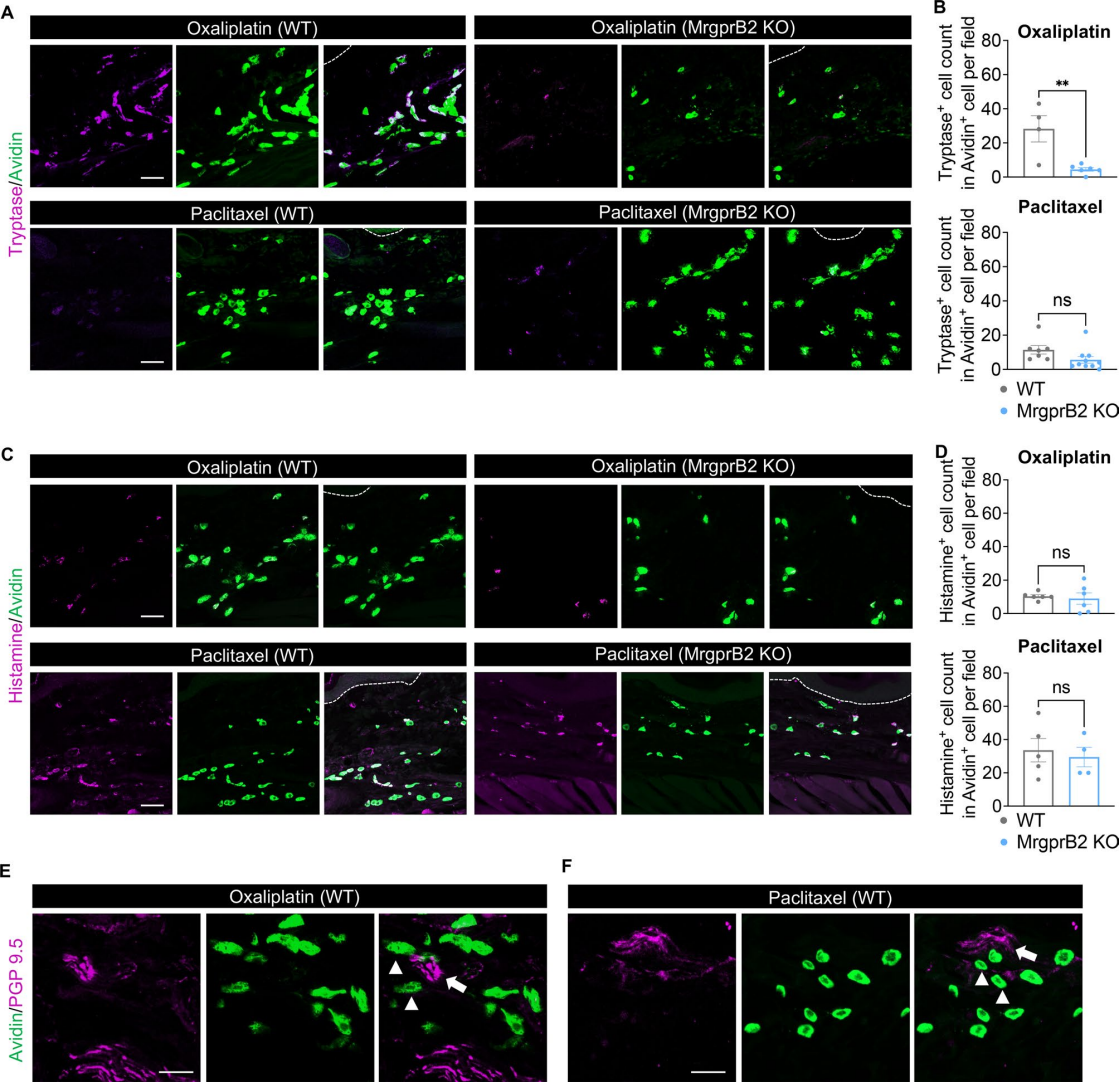
MrgprB2 deletion selectively prevented oxaliplatin-induced tryptase upregulation but did not alter paclitaxel-evoked histamine elevation in mast cells. (**A**) Representative confocal immunofluorescence images of hindpaw skin sections stained for tryptase (magenta) and avidin (green) in wild-type (WT) or MrgprB2 mice treated with oxaliplatin or paclitaxel. Scale bar, 100 μm. (**B**) Quantification of tryptase+ cells per field: significantly decreased in MrgprB2 KO mice compared to WT mice after oxaliplatin treatment. However, the numbers were comparable between genotypes after paclitaxel treatment. (**C**) Representative confocal immunofluorescence images of hindpaw skin sections stained for histamine (magenta) and avidin (green) in WT and MrgprB2 KO mice treated with oxaliplatin or paclitaxel. Scale bar, 200 μm. (**D**) Quantification of histamine^+^ cells per field showed no significant difference between genotypes for either treatment. B, D: data are mean ± SEM. **p < 0.01, one-way ANOVA with Tukey’s multiple comparisons test (*n* = 4/group). (**E, F**) Representative confocal image of mouse hindpaw sections stained for avidin (green, arrows) and peripheral afferents (magenta, PDG9.5, arrowheads) after (E) oxaliplatin or (F) paclitaxel treatment. Scale bar (E, F), 100 μm

Quantification showed that the upregulation of tryptase^+^ MCs in the hindpaw skin of WT mice after oxaliplatin treatment was significantly attenuated in MrgprB2 KO mice (Fig. 7A, B). Both genotypes showed similarly low numbers of tryptase^+^ MCs in the skin after paclitaxel treatment.

The number of histamine^+^ MCs remained low in both WT and MrgprB2 KO mice following oxaliplatin treatment (Fig. 7C, D). Whereas paclitaxel produced a similar increase in the number of histamine^+^ MCs in both genotypes. These findings indicate that MrgprB2 is required for oxaliplatin-induced tryptase upregulation in MCs, but not for the paclitaxel-induced histamine elevation [34]. PGP 9.5 is a pan-neuronal marker to visualize cutaneous nerve fibers [54, 55]. Confocal imaging revealed close spatial proximity between some avidin^+^ MCs and PGP 9.5-labeled peripheral afferents in hindpaw skin of WT mice after oxaliplatin and paclitaxel treatment (Fig. 7E, F).

## Discussion

Although CIPN pain is a major clinical challenge, increasing evidence suggests that different chemotherapeutic classes engage partially distinct pathogenic mechanisms [7, 18, 56, 57]. Neuroimmune interactions have emerged as a critical contributor to pain sensitization [11, 16], but the receptor pathways linking mast cells to CIPN pain across drug classes remain poorly defined. Our study identifies a novel receptor-level divergence in MC engagement between oxaliplatin and paclitaxel, and supports a selective requirement for MrgprB2 in oxaliplatin-associated MC activation in the skin and CIPN pain-like behavior in mice.

We found that oxaliplatin, but not paclitaxel, produced a robust increase of tryptase staining in skin MCs of WT mice, whereas this elevation was significantly reduced in MrgprB2 KO mice. Therefore, oxaliplatin may selectively produce an MC activation profile enriched for tryptase release and dependent on MrgprB2. Importantly, both genetic deletion and pharmacological inhibition of MrgprB2 selectively attenuated oxaliplatin-induced cold and mechanical hypersensitivity without altering paclitaxel-induced pain. Pharmacologically, osthole provided an independent means of testing this pathway in vivo. Prior work supports its ability to inhibit MrgprB2/X2-mediated MC activation, although it is not receptor-selective, and off-target actions, including calcium channel effects, cannot be excluded [58]. In our study, however, its analgesic effect was lost in MrgprB2 KO mice, making a purely nonspecific explanation less likely and supporting the view that oxaliplatin-associated pain relief by osthole depends primarily on the presence of MrgprB2.

Our side-by-side comparison of oxaliplatin and paclitaxel is particularly informative in this context. By showing that MrgprB2 deletion and pharmacological inhibition attenuated the oxaliplatin phenotype but not the paclitaxel phenotype, the present study suggests that CIPN pain can arise through mechanistically distinct neuroimmune pathways rather than through a single shared mast cell mechanism. MrgprB2/X2 is better understood as a context-dependent effector that helps distinguish platinum-associated from taxane-associated pain pathways. Future studies using more selective MrgprX2 antagonists, including newer tool compounds developed for this pathway, will be important for testing this idea in humanized and disease-relevant systems.

Notably, in vitro, neither oxaliplatin nor paclitaxel directly activated WT mouse MCs, nor did either drug sensitize MCs or enhance their calcium response to Substance P and S1P. The cell viability assays further support the interpretation of these negative findings. Under assay-matched conditions, neither oxaliplatin nor paclitaxel caused a loss of MC viability at the concentrations used in the calcium imaging and β-hexosaminidase experiments. These results make it unlikely that the absence of direct MC degranulation or calcium responses simply reflected nonspecific cytotoxicity.

In vivo, the activation and degranulation of MCs can be triggered by the mediators released from sensory neurons and other local cells [16, 27], and our findings are therefore more consistent with an indirect neuro-immune mechanism operating in the skin than with direct activation of MCs by oxaliplatin or paclitaxel under the conditions tested. Within this framework, Substance P is more plausibly interpreted as a downstream amplifier than as the initiating signal in the oxaliplatin model. This distinction is important because prior work has shown that paclitaxel can promote Substance P release from cultured rat DRG neurons, whereas oxaliplatin does not [59], arguing against a simple model in which oxaliplatin directly induces neuronal Substance P release that then activates MrgprB2on MCs. A more cautious interpretation of our data is that oxaliplatin may first perturb sensory neurons or cells within the local tissue microenvironment via an as-yet-unknown upstream signal, leading to initial MrgprB2-dependent MC activation and tryptase release. Tryptase could then act on neuronal PAR2, promoting secondary neuropeptide release, including Substance P, which may amplify local MC-neuron crosstalk rather than initiate it [27]. This sequence is compatible with prior platinum-related work and with the known ability of Substance P to activate MrgprB2, but it remains a working model rather than a demonstrated mechanism in the present study.

Consistent with this interpretation, we observed robust tryptase release in oxaliplatin-treated skin, and this response was MrgprB2-dependent. Anatomically, confocal imaging revealed close spatial proximity between the recruited avidin^+^ MCs and PGP9.5-labeled peripheral afferents in hindpaw skin after oxaliplatin and paclitaxel treatment, providing the anatomical substrate consistent with local neuro-immune crosstalk. At the same time, these observations should be interpreted as compatible with, rather than proof of, a reciprocal feed-forward loop. Other endogenous MrgprB2/X2 ligands (e.g., PAMP-12, LL-37) may also be plausible depending on tissue context, but their contribution to oxaliplatin-induced CIPN remains unknown.

This interpretation may also help explain the divergence between oxaliplatin and paclitaxel in our study. Although paclitaxel has been reported to increase Substance P release [59], MrgprB2 deletion or pharmacological inhibition did not attenuate paclitaxel-induced pain here, suggesting that Substance P–MrgprB2 signaling is unlikely to be the dominant driver of the paclitaxel phenotype in our model. This argues against Substance P–MrgprB2 signaling as a dominant organizing principle for CIPN pain in general. Instead, paclitaxel has strong support for engaging TLR4-dependent inflammatory signaling in DRG and associated immune cells [26], which may better account for its pain phenotype independently of MrgprB2. Together, these findings suggest that oxaliplatin- and paclitaxel-induced CIPN engage overlapping but non-identical neuroimmune programs. More broadly, they underscore the importance of classifying CIPN pain by underlying biological pathways rather than by symptomatology alone, and support the idea that MrgprB2 may help distinguish platinum-associated from taxane-associated pain mechanisms. Given the functional conservation between murine MrgprB2 and human MrgprX2, and the growing interest in MrgprX2-directed therapeutics [16, 18, 19, 24, 41, 60, 61], our data suggest that targeting this axis may be more relevant to platinum-associated pain mechanisms than to taxane-associated ones.

Our study has some limitations. 1) Most importantly, although the behavioral, histological, and pharmacological data support a selective requirement for MrgprB2 in oxaliplatin-associated pain, the upstream neuronal and/or non-neuronal trigger remains unresolved. 2) Oxaliplatin and paclitaxel models differ substantially in induction duration. Although tissues were collected at the maintenance phase of pain-like behavior in each paradigm, rather than at identical elapsed times after treatment initiation, this difference may influence the local inflammatory microenvironment, MC phenotype, and mediator release profile at the time of analysis. Since MC are known to deploy distinct secretory programs depending on stimulus context, the relative enrichment of tryptase- and histamine-positive mast cells observed here should be interpreted with this temporal difference in mind, rather than as a strictly time-independent drug-specific signature [25]. 3) Although intraepidermal nerve fiber (IENF) loss is a hallmark of late-stage CIPN, our study focused on pain behaviors in which neuroinflammation plays a dominant role, rather than on structural neurodegeneration (e.g., IENF loss quantified by PGP9.5 staining) caused by CIPN. The fact that both MrgprB2 KO and blockade reversed oxaliplatin-associated pain suggests that targeting this functional neuro-immune regulator may provide effective pain relief, potentially independent of structural regeneration. 4) Our analysis was limited to oxaliplatin and paclitaxel. Extending this mechanistic framework to other chemotherapeutic classes, including cisplatin, carboplatin, vincristine, and bortezomib, will be essential to define their position along the MrgprB2-dependent versus MrgprB2-independent spectrum. 5) Although we did not observe a significant sex-dependent divergence that altered the main interpretation of the pooled results, larger studies designed a priori around sex as a biological variable will be needed to determine whether the magnitude, timing, or downstream immune features of MrgprB2-dependent pain signaling differ between males and females. 6) We also cannot completely exclude minor nonspecific contributions from vehicle components, particularly because paclitaxel required a Cremophor EL/ethanol-based formulation. However, all in vitro and in vivo findings were interpreted relative to matched vehicle controls, and the consistency between the pharmacological and genetic results in the oxaliplatin model argues against vehicle effects being the primary explanation for our conclusions.

In summary, the current study suggests that oxaliplatin, but not paclitaxel, selectively engages an MrgprB2-dependent pathway in vivo that contributes to CIPN pain. By defining a receptor-level divergence between platinum- and taxane-associated neuroimmune programs, our findings support a framework in which CIPN is better understood as a group of related but mechanistically distinct pain states. This MrgprB2-dependent and drug-specific neuroimmune architecture offers a promising avenue for developing targeted therapies that may alleviate pain in patients receiving platinum-based chemotherapy. Further translational progress will require validation in a humanized MrgprX2 mouse model of CIPN to assess clinically relevant MrgprX2 antagonists, as well as studies in cancer-bearing models to ensure that MrgprX2 blockade does not impair the chemotherapeutic efficacy or cause side effects.

## Electronic supplementary material

Below is the link to the electronic supplementary material.

Supplementary Material 1

## Data Availability

The datasets used and/or analyzed during the current study are available from the corresponding author on reasonable request.
