# Supplementary material for: Mast cell-specific receptor MrgprB2 selectively mediates oxaliplatin-induced neuropathic pain
Source: J Transl Med. 2026 May 23;24:938. doi: 10.1186/s12967-026-08285-w (PMC13386573; doi:10.1186/s12967-026-08285-w)
Supplement: Supplementary file 1 — Supplementary Material 1 [file 12967_2026_8285_MOESM1_ESM.docx]

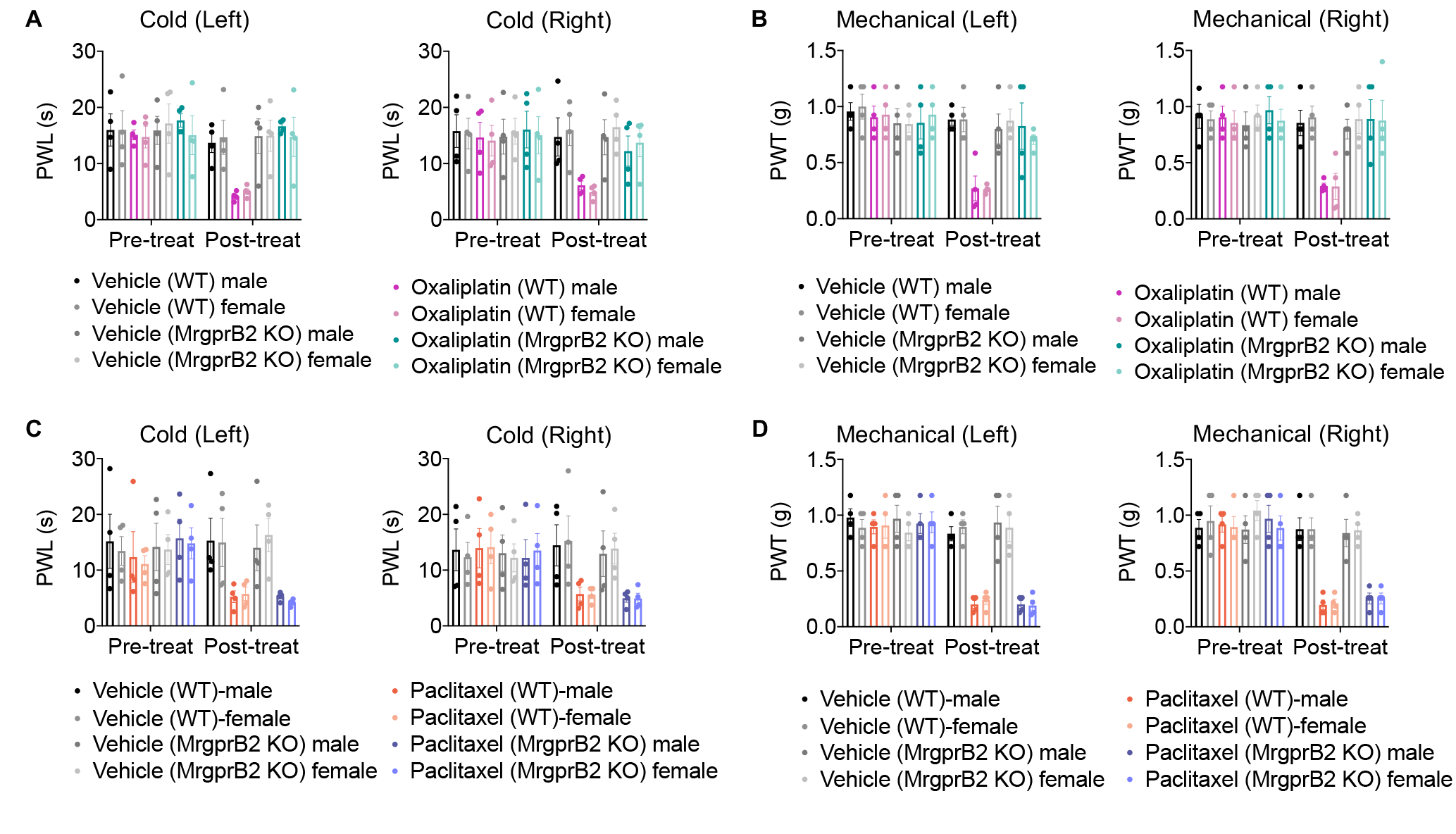


**Figure S1. Sex-disaggregated analysis of oxaliplatin- and paclitaxel-induced pain in wildtype (WT) and MrgprB2 KO mice.** **(A, B)** Oxaliplatin-induced cold (A) and mechanical (B) hypersensitivity are shown separately in male and female mice in each genotype. **(C, D)** Paclitaxel-induced cold (C) and mechanical (D) hypersensitivity are shown separately in male and female mice in each genotype. Data are shown as mean ± SEM, n=4/group.


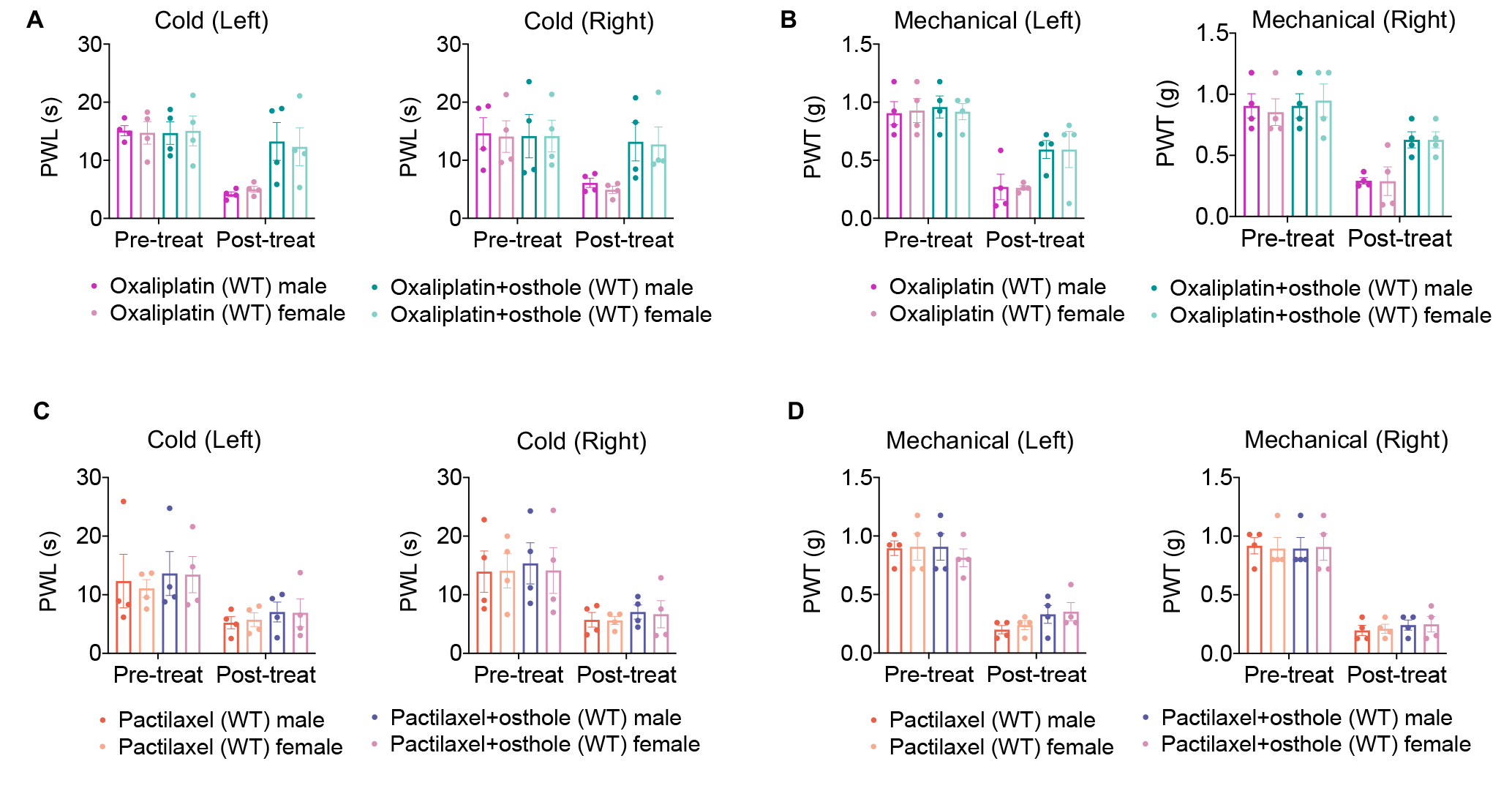


**Figure S2. Sex-disaggregated analysis of the effects of pharmacological MrgprB2 inhibition on oxaliplatin- and paclitaxel-induced pain in wildtype (WT) mice.** **(A, B)** Oxaliplatin-induced cold (A) and mechanical (B) hypersensitivity are shown separately in male and female WT mice. **(C, D)** Paclitaxel-induced cold (C) and mechanical (D) hypersensitivity are shown separately in male and female WT mice. Data are shown as mean ± SEM, n=4/group.


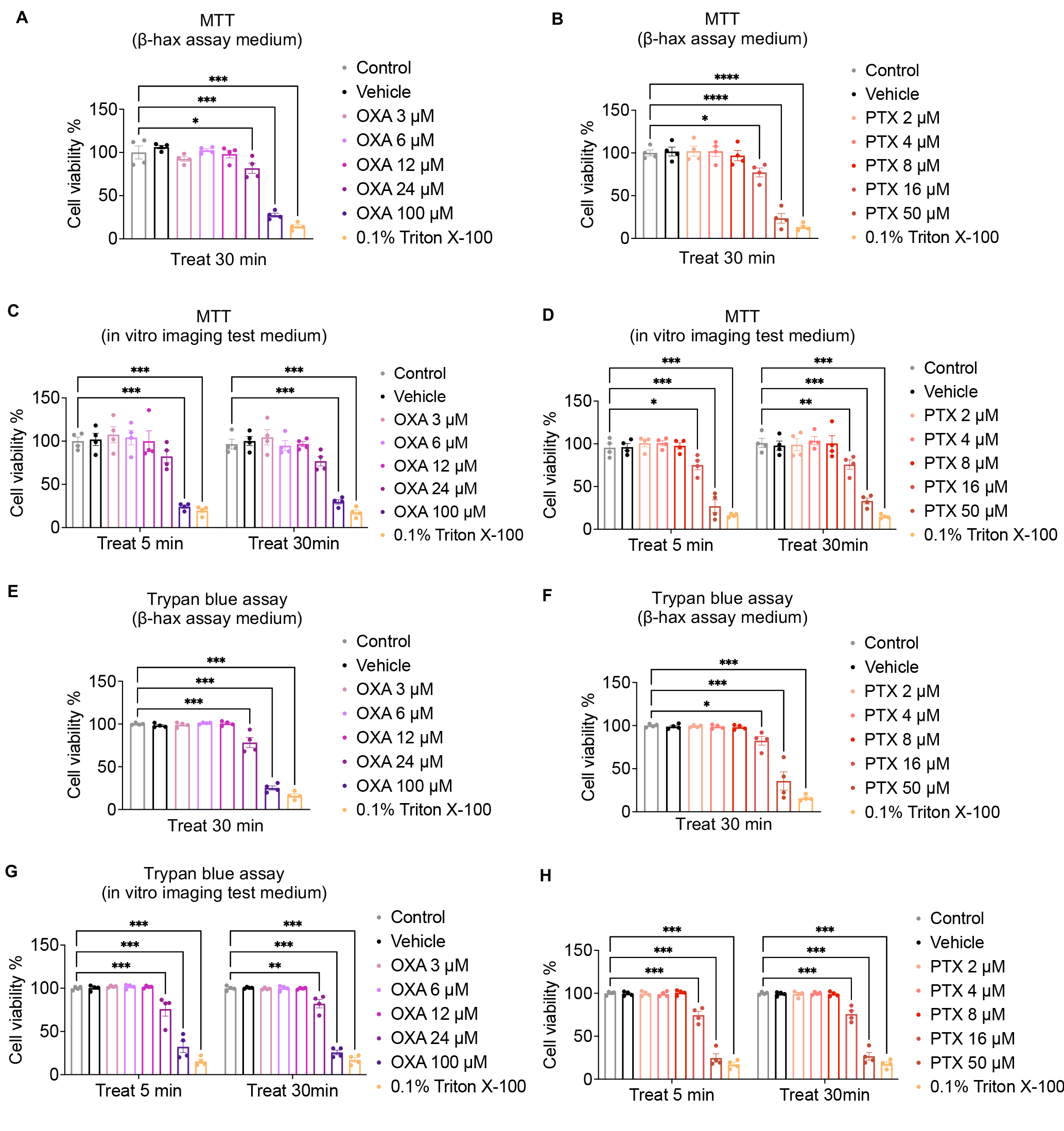


**Figure S3. Viability of cultured mast cells under assay-matched conditions for β-hexosaminidase experiments and calcium imaging.** **(A–D)** MTT assay. **(E–H)** Trypan blue exclusion assay. (A, C, E, G) Oxaliplatin conditions, including control, vehicle-matched control, oxaliplatin at the indicated concentrations, and 0.1% Triton X-100 as a positive control for loss of viability. (B, D, F, H) Paclitaxel conditions, including control, vehicle-matched control, paclitaxel at the indicated concentrations, and 0.1% Triton X-100 as a positive control for loss of viability.
(A, B, E, F) Assays performed under medium conditions matched to the β-hexosaminidase degranulation assay. **(C, D, G, H)** Assays performed under medium conditions matched to the *in vitro* calcium imaging assay. For MTT analysis, all values were background-corrected by subtracting blank wells and then normalized to the corresponding control group. For trypan blue analysis, viability was calculated as the percentage of unstained cells among the total number of cells counted and normalized to the corresponding control group. Under all assay-matched conditions, oxaliplatin and paclitaxel at the working concentrations used in the functional experiments, together with 0.1% Triton X-100, served as a positive control. Data are shown as mean ± SEM, n = 4 repetitions/group. One-way ANOVA (A, B, E, F) or two-way ANOVA (C, D, G, H) with Dunnett’s multiple comparisons tests. Statistical comparisons were made versus the corresponding control group. **P* < 0.05, ***P* < 0.01, ****P* < 0.001.
